# Ultrafast electronic state conversion at room temperature utilizing hidden state in cuprate ladder system

**DOI:** 10.1038/ncomms9519

**Published:** 2015-10-20

**Authors:** R. Fukaya, Y. Okimoto, M. Kunitomo, K. Onda, T. Ishikawa, S. Koshihara, H. Hashimoto, S. Ishihara, A. Isayama, H. Yui, T. Sasagawa

**Affiliations:** 1 CREST, JST, Kawaguchi, Saitama 332-0012, Japan; 2 Department of Chemistry and Materials Science, Tokyo Institute of Technology, Meguro, Tokyo 152-8551, Japan; 3 Interactive Research Center of Science, Tokyo Institute of Technology, Yokohama, Kanagawa 226-8502, Japan; 4 PRESTO, JST, Kawaguchi, Saitama 332-0012, Japan; 5 Department of Physics, Tohoku University, Sendai 980-8578, Japan; 6 Materials and Structures Laboratory, Tokyo Institute of Technology, Yokohama, Kanagawa 226-8503, Japan

## Abstract

Photo-control of material properties on femto- (10^−15^) and pico- (10^−12^) second timescales at room temperature has been a long-sought goal of materials science. Here we demonstrate a unique ultrafast conversion between the metallic and insulating state and the emergence of a hidden insulating state by tuning the carrier coherence in a wide temperature range in the two-leg ladder superconductor Sr_14-x_Ca_x_Cu_24_O_41_ through femtosecond time-resolved reflection spectroscopy. We also propose a theoretical scenario that can explain the experimental results. The calculations indicate that the holes injected by the ultrashort light reduce the coherence among the inherent hole pairs and result in suppression of conductivity, which is opposite to the conventional photocarrier-doping mechanism. By using trains of ultrashort laser pulses, we successively tune the carrier coherence to within 1 picosecond. Control of hole-pair coherence is shown to be a realistic strategy for tuning the electronic state on ultrafast timescales at room temperature.

## Abstract (web summary)

Controlling material properties on sub-picosecond scales using photons could allow for ultrafast optoelectronic devices. Here, the authors propose an ultrafast photoinduced metal-to-insulator transition in a two-leg ladder cuprate superconductor based on time-resolved reflection spectroscopy.

## Graphical abstract

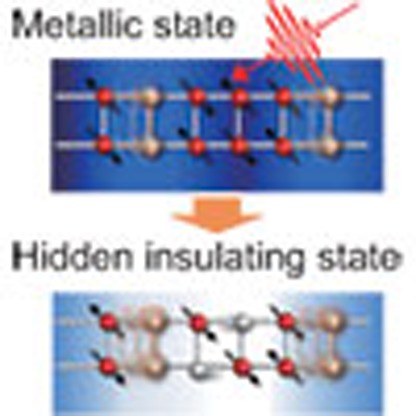

Photo-control of electronic and optical properties on femtosecond (10^−15^ s:fs) and picosecond (10^−12^ s:ps) timescales at room temperature has been a long-sought goal of materials science as it would enable the creation of devices for ultrafast and efficient optical and/or electronic signal processing. In particular, intensive studies have been devoted to photoinduced phase switching in strongly correlated matter, that is, matter with couplings among the charge, spin and orbital degrees of freedom. Here gigantic changes in the optical, conductive and magnetic properties can be expected as a result of ultrafast conversion from insulating to metallic (I to M) electronic states by virtue of photo-carrier injection and/or orbital modifications^1^,^2^,^3^,^4^,^5^. Carrier doping by photoexcitation beyond the energy gap is a reasonable idea for ultrafast photonic control of the electronic properties and inducing ultrafast gigantic optical responses, because the natural expectation is that photocarriers would be generated on fs to ps orders through direct photo-modulation of the charge degree of freedom in condensed matter systems^1^,^2^,^3^,^4^,^5^,^6^. Most of the recent studies aiming at triggering ultrafast conversion from the insulator to metallic state in the low temperature region are based on the idea that photocarriers can be injected into condensed matter.

Despite the existence of photodoping via direct carrier injection and/or orbital structure modification, materials research has faced a difficult question of whether bi-directional and ultrafast conversion between insulator and metallic states can be realized optically at high operating temperatures. The prevailing idea would make it difficult to perform the opposite, that is, M to I conversion by photo-modulation of the conventional degrees of freedom, because photoexcitation, including photocarrier doping, generally leads to an increase in the carrier number and results in an enhancement of the metallicity of the system.

The target material is a two-leg ladder superconducting cuprate, Sr_14-x_Ca_x_Cu_24_O_41_, constructed from one-dimensional edge-sharing CuO_2_ chains (Fig. 1a) and two-leg corner-sharing Cu_2_O_3_ ladders (Fig. 1b). The sublattice layers are alternately stacked on and separated from each other by Sr/Ca atom layers (Fig. 1c)^7^,^8^,^9^. The nominal valence of the Cu ions is +2.25, as a result of inherent hole doping. The electronic properties are determined by holes that are doped in the ladder; most of the holes in the chains are localized^10^. Dielectric measurements on the parent compound, Sr_14_Cu_24_O_41_ (SCO), revealed the existence of a charge-density wave below about 210 K (=*T*_c_) with an energy gap of about 130 meV (ref. 11). The proposed order pattern of the charge-density wave in the ladder is such that the holes are paired along the rungs and the pairs appear every five ladder units along the legs, whereas they are disordered in the normal insulating state above *T*_c_ (refs 12, 13). Isovalent substitution of Ca for Sr ions changes the electronic properties around room temperature from insulative to metallic by redistributing holes between the chains and ladders, and this system, when it has a high-Ca composition at *x*>10, shows superconductivity below 12 K under an external pressure of 3–8 GPa (refs 14, 15).

Although the Sr_14-x_Ca_x_Cu_24_O_41_ system has intensively been investigated as a high-temperature superconductor^7^,^10^,^11^,^12^,^13^,^14^,^15^, the nature of its carrier conductivity is still unclear. Theoretical studies predict that hole pairs are responsible for the superconductivity^16^, implying that the coherence among hole pairs plays an essential role in carrier conductivity, as depicted in Fig. 1d. Thus, the paired state is a key to understanding the electronic state of the ladder-type superconductor. To confirm this theoretical prediction, one must look for an external stimulation that can be used to tune the hole-pair coherence and be a new way of insulating-metallic state (I–M) control.

Here we describe experimental and theoretical results showing that an ultrafast I to M and M to I electronic state conversion can be achieved even at room temperature by tuning the carrier coherence in a two-leg ladder superconductor Sr_14-x_Ca_x_Cu_24_O_41_ by using femtosecond time-resolved reflection spectroscopy over a wide temperature range. The experimental results show photoinduced I to M and M to I conversions as well as the appearance of a transient insulating state which never appears in the ground state (that is, a hidden state^6^). The theoretical study reveals that the holes injected by ultrafast light pulses reduce the coherence among the inherent hole pairs and result in suppression of conductivity, which is opposite to the conventional photocarrier doping mechanism. In addition, by using trains of ultrafast laser pulses, we were able to perform successive transient I–M conversion within 1 ps after excitation by virtue of the appearance of the hidden insulating state. Control of hole-pair coherence is a new and realistic strategy for tuning the electronic state on ultrafast timescales at room temperature.

## Results

### Transient reflectivity and optical conductivity spectra

Our experimental study involved time-resolved measurements using a pump–probe technique (see Methods). The parent insulating compound SCO was measured in at 100 K and at 290 K, and the metallic compound Sr_4_Ca_10_Cu_24_O_41_ (SCCO) was measured at room temperature (290 K) while being excited by laser light with a 120 fs pulse width at a photon energy of 1.58 eV, which can excite the Cu–O charge transfer transition in the ladder^17^. As discussed in our previous report^18^, a large Drude response appears within 1 ps after the photoexcitation of SCO at 100 K (see the red circles in Supplementary Fig. 1a); the estimated Drude weight is comparable to that of the hole-doped metallic state, indicating a photoinduced I to M transition^18^. In addition, a comparable amount of the Drude weight appears after the photoexcitation even at room temperature (see the green squares in Supplementary Fig. 1a), indicating the production of the similar photoinduced metallic state in wide temperature range.

The prevailing ideas about semiconductors would lead one to think that photoexcitation of SCCO, which is a metallic compound, will increase the carrier density and simply result in an enhancement of metallicity as shown in Fig. 2a. However, unexpectedly, an instantaneous suppression response was observed within 200 fs after the photoexcitation (see the shaded area in Fig. 2b). Only well after (>1 ps) the excitation did the Δ*R*/*R* signal, where Δ*R* is the differential reflectivity between before and after the photoexcitation and *R* is the static reflectivity before the photoexcitation, change to an enhanced response that is consistent with the enhancement of metallicity that occurs in SCO shown in Supplementary Fig. 1a,. This transient response hardly shows temperature dependence.

The reflectivity spectrum just after photoexcitation, as estimated from Δ*R*/*R* measured at various probe photon energies, is shown in Fig. 3a (circles). The inherent Drude response exhibited in the static reflectivity spectrum (solid) is suppressed just after the photoexcitation, and this response is opposite to that in SCO. After 2 ps, the Drude response is enhanced, similar to that in SCO, as shown in Supplementary Fig. 2. To quantitatively discuss the suppression of reflectance, we calculated the optical conductivity by performing a Kramers–Kronig (K–K) analysis on the transient reflectivity spectra taking into account of the spatially inhomogeneous distribution of the photoinduced part after the pumping^19^,^20^. In the K–K analysis, we assumed constant reflectivity below 0.1 eV and connected the obtained transient reflectivity with the reflectivity before the photoexcitation above 4.0 eV on the ground that the photoinduced reflectance change is quite small above 4.0 eV. The penetration depth is about 80 nm at the pump wavelength. The detail of the analysis is described in Methods. Figure 3b shows the calculated optical conductivity just after the photoexcitation, together with the optical conductivity in the original metallic state. The spectral weight of the conductivity is suppressed below 0.8 eV while increased above 0.8 eV after the photoexcitation. By integrating the conductivity data, we can estimate the spectral weight that corresponds to the effective electron number. The estimated effective electron number is 0.121 before the photoexcitation and 0.107 just after the photoexcitation. This spectral change clearly indicates the suppression of the Drude weight, that is, photoinduced M to I conversion, and it is also found by the analysis assuming the situation of the spatially homogeneous distribution (Supplementary Fig. 2, Supplementary Table 1 and Supplementary Note 1). Therefore, the insulating and metallic states of the ladder system surely have different photoinduced phenomena, indicating that the direction of the photonic change strongly depends on the inherent carrier density before the photoexcitation.

### Photonic control of direction in I–M state conversion

In terms of the phototuning of I–M conversion direction on sub-ps timescales, we performed sequential photoexcitation measurements of SCO, as schematically illustrated in Fig. 4a, and demonstrated ultrafast conversion control by phototuning the carrier density. The carrier density in the photoinduced metallic state after the first pumping can be controlled by the pump fluence^18^ and then we irradiated the sample with a second pump pulse after a finite interval (Δ*t*).

The fluence dependence of Δ*R*/*R* probed at 0.5 eV at 1 ps with one pump (single) pulse excitation is shown in Fig. 4b. The increase of the reflectivity change corresponds to the increase of the Drude response, that is, the carrier density. The carrier density in Sr_5_Ca_9_Cu_24_O_41_ compound on the phase boundary of the I–M transition is created at ca. 8 mJ cm^−2^ shown in the dashed line in the figure, estimated form the Drude weight (Supplementary Note 2). With further increase in the fluence, the signal gradually increases and finally saturates at about 20 mJ cm^−2^ and the sample is damaged by the pump pulses above 25 mJ cm^−2^. With such a single excitation, the ultrafast reduction of reflectivity seen in SCCO has never been observed even above the saturation threshold fluence.

The experimental Δ*R*/*R* time profiles with two pump (double) pulse excitation are shown in Fig. 4d. The experimental conditions of probing energy and temperature are the same as in Fig. 4b. The first-pump fluence was varied, while the second one was fixed. The Δ*R*/*R* signals just after the first photoexcitation at *t*_1st_ rapidly increased with increasing fluence, reflecting the enhancement of the carrier density^18^. The second photoexcitation, 1 ps (=*t*_2nd_) after the first excitation, induced a reflectance change that strongly depended on the first pump's fluence. Figure 4e shows differential Δ*R*/*R* profiles between the double and single photoexcitation profiles at 100 K. The profile at the lowest fluence is similar to the single photoexcitation response (see the solid line in Fig. 2a), indicating photoexcitation of the rest of the insulating state by the second pulse. Then, the signal decreases with increasing fluence, and a profile almost identical to that of SCCO appears (Fig. 2b). The fluence dependence at *t*_2nd_ is plotted in Fig. 4c. The deviation of the Δ*R*/*R* value for the single excitation (horizontal dotted line) that resulted from the ultrafast suppression response due to the second excitation, appears at around the corresponding fluence of the carrier density in Sr_5_Ca_9_Cu_24_O_41_, and increases with increasing fluence. The similar fluence dependence of the time profiles and suppression response also appeared at room temperature, as shown in Fig. 4f,g. These experimental results clearly indicate that I–M conversion direction can be tuned simply by using light within 1 ps and over a wide temperature range and that the spectroscopic nature of the photoinduced metallic state of SCCO is similar to that of the chemically hole-doped metallic state of SCCO.

## Discussion

To understand the observed unexpected response of SCCO and the I–M conversion by two pump pulses, we propose a possible scenario based on the theoretical analyses of the photoexcited state in ladder cuprates. We set up a two-leg ladder Hubbard model that takes into account electron hopping matrix elements *t*_‖_(legs) and *t*_⊥_(rungs) with an isotropic condition, *t*_‖_=*t*_⊥_(∼0.3–0.5 eV in ladder cuprates), and an on-site Coulomb interaction *U* (refs 21, 22). Figure 3c,d show the reflectivity and optical conductivity spectra before (black) and after (red) the photoexcitation theoretically predicted in the doped metallic system for SCCO. It is noted that the pump photon energy is tuned at the inter-site charge transfer energy, in the same way to the experiments. Prominent low energy components observed in the optical conductivity spectra correspond to the Drude response in the finite-sized cluster calculation^23^. In the open boundary condition adopted in the calculation shown in Fig. 3c,d, the Drude weight can be extracted from the so-called ‘Drude precursor' peak at finite lowest-excitation energy (corresponding to 0.6 *t*_‖_ in Fig. 3d), which is limited due to the largest length scale of the cluster. This lowest excitation energy is confirmed to be reduced with increasing the cluster size, as shown in Supplementary Fig. 3. Note that the calculated low-energy optical response reflecting a metallic state is remarkably suppressed after the photoexcitation, opposite to the enhancement of the response due to the photoinduced I to M conversion in the calculations for SCO (Supplementary Fig. 1b). Both the calculated reflectivity and optical conductivity spectra are qualitatively consistent with the experimental spectra in Fig. 3a,b. The photoinduced suppression of the metallic state is linked to the two-leg ladder lattice, because the theoretically calculated changes in the Drude weight *D*(*t*) due to photoexcitation are significantly modified by the anisotropy of the electron hopping matrix elements, *r*_*t*_≡*t*_⊥_/*t*_‖_ (Fig. 5). The theoretical results of the sequential photoexcitation shown in Fig. 4h,i also reproduce the experimental results at 100 K and 290 K shown in Fig. 4d–g, although there is a possibility of increasing in the relaxation rates due to the lattice and orbital degrees of freedom, which are not taken into account in the theoretical model.

Equilibrium studies of the Hubbard ladder system have shown that singlet hole pairs play a key role through their unique electronic structure^21^,^24^. The present calculation explores the following singlet pair-field correlation function in the photoexcited transient state:


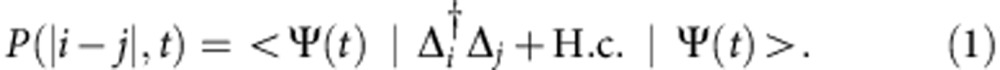


**Figure 1:**
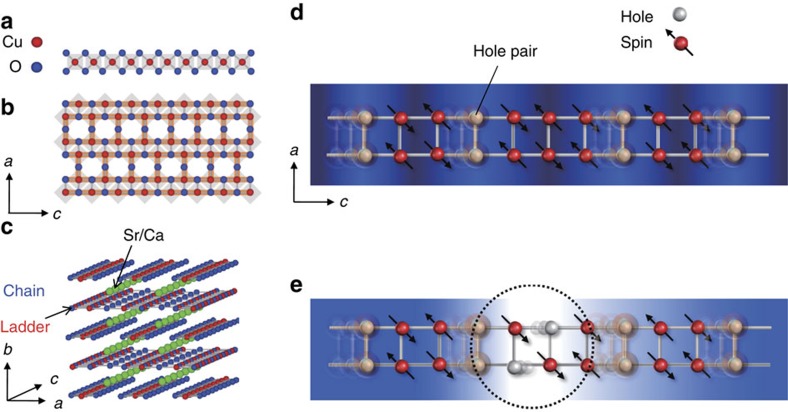
Structure of Sr_14-x_Ca_x_Cu_24_O_41_ and hole states in ladder sublattice. Schematic structures of (**a**) CuO_2_ chain and (**b**) Cu_2_O_3_ ladder sublattices. (**c**) Three dimensional view of Sr_14-x_Ca_x_Cu_24_O_41_ structure. Red, blue and green circles respectively indicate Cu, O and Sr or Ca ions. Shaded squares in **a** and **b** indicate CuO_2_ sites. Illustrations of (**d**) hole-doped, and (**e**) photoinduced insulating states in the ladder sublattices. Grey circles and red circles with arrows indicate Cu ions with a hole and spin, respectively. Shaded circles on the Cu ions with a hole represent hole pairs. The hole pairs are moved coherently along *c*-direction. Colour gradients denote the wave function of the hole pair. The singlet pair-field correlation is illustrated by the overlapping of the wave functions, and it collapses as a result of the photo-injected holes shown in the dotted circle area in **e**.

**Figure 2:**
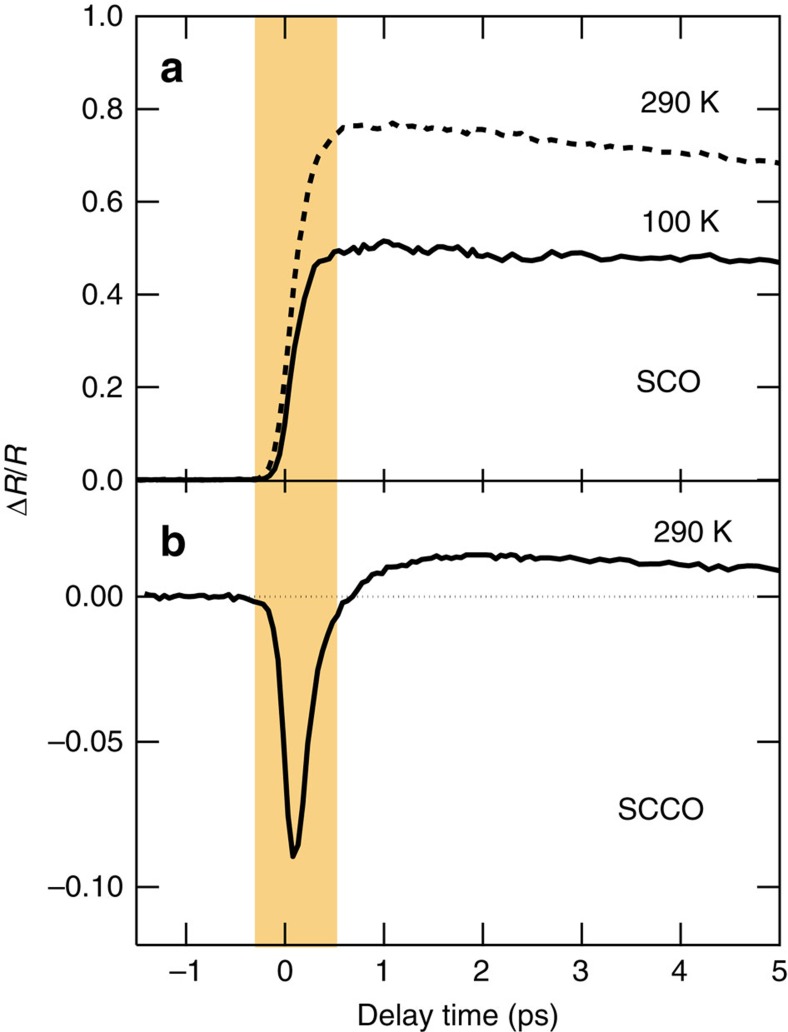
Time profiles of reflectivity change. Time profiles of reflectivity change ΔR/R at 0.5 eV in (**a**) SCO at 100 K (solid line) and 290 K (dashed line) and in (**b**) SCCO at 290 K. The excitation fluences are about 13 mJ cm^−2^ in SCO and about 8.2 mJ cm^−2^ in SCCO. The shaded area approximately corresponds to the timescale of the theoretical calculation results.

**Figure 3:**
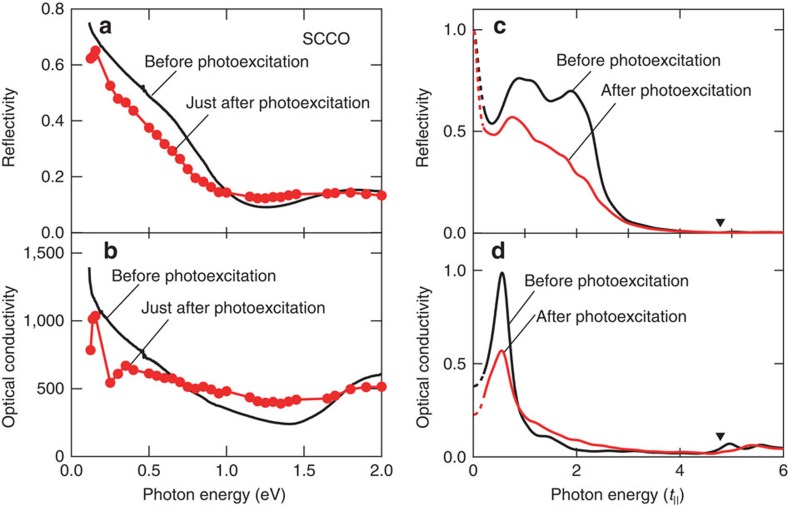
Transient Drude responses in metallic states. (**a**) Reflectivity spectra with polarization of *E*||*c* before and after photoexcitation on SCCO at 290 K. The excitation fluence is about 8.6 mJ cm^−2^ in SCCO. (**b**) Optical conductivity *σ* (Ω^−1^cm^−1^) spectra obtained from K–K analysis of reflectivity spectra before and after photoexcitation on SCCO at 290 K. The K–K analysis on the transient reflectivity spectrum takes into account of the spatially inhomogeneous distribution of the photoinduced part after the pumping^19^,^20^. The solid lines and circles in **a** and **b** indicates the reflectivity before and just after the photoexcitation, respectively. (**c**,**d**) Calculated reflectivity and optical conductivity spectra with polarization along the leg before (*t*<<0) and after (*t*=20/*t*_‖_) photoexcitation under metallic (*n*=0.833) condition. A finite-sized cluster of 2 × 6 sites with open boundary conditions along the legs is used. The on-site Coulomb interaction is *U*/*t*_‖_=6. Bold inverted triangles indicate the pump-photon energies. Negative values in the spectra after the photoexcitation imply optical emission. The dashed lines below about 0.2 *t*_‖_ are reference data due to the calculation size effect.

**Figure 4:**
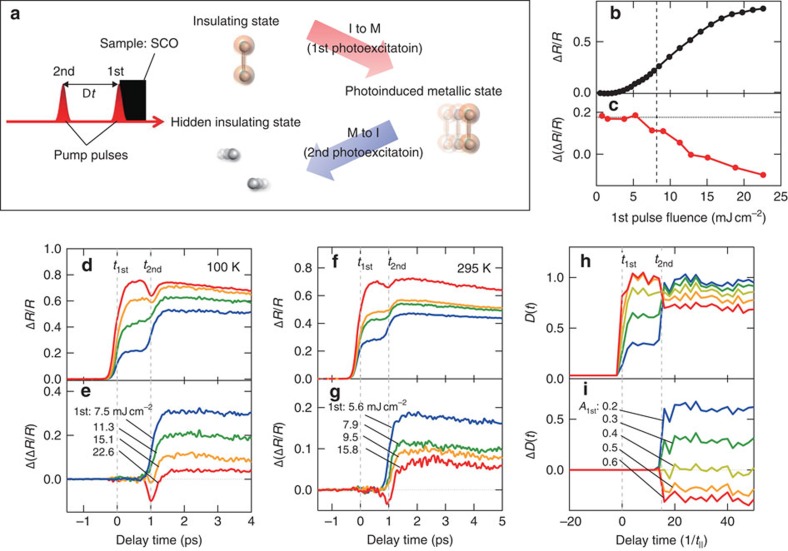
Photonic control of direction in insulating-metallic state conversion by sequential photoexcitation. (**a**) Measurement scheme of sequential photoexcitation. Δ*t* (=*t*_2st_-*t*_1nd_) is the time interval between the first and second pulses. Red arrow indicates the incident direction of the pump pulses. The SCO indicated by the black square is irradiated with the first and second pulses at 0 ps (=*t*_1st_) and 1 ps after (=*t*_2nd_). After the irradiation by the first pulse, localized hole-pairs in the insulating state move coherently, resulting in an increase in the carrier density (I to M conversion, see the red wide arrow). After the second pulse, the single holes are created and the carrier conductivity in the photoinduced metallic state is suppressed because the hole-pair coherence collapses (M to I conversion, see the blue wide arrow). (**b**) Fluence dependence of Δ*R*/*R* probed at 0.5 eV at 1 ps with one pump (single) pulse excitation and (**c**) first pump pulse fluence dependence of differential Δ*R*/*R* (Δ(Δ*R*/*R*)) just after second excitation (=*t*_2nd_) with two pump (double) pulse excitation in SCO at 100 K. The horizontal dotted line in **c** indicates the value of Δ*R*/*R* at 1 ps for a single excitation at the same second pump pulse fluence in the two pump excitation measurement. The vertical dashed line indicates the corresponding fluence of the carrier density in Sr_5_Ca_9_Cu_24_O_41_ compound on the phase boundary of the I–M transition, as estimated from the Drude weight. (**d**,**f**) Time profiles of Δ*R*/*R* probed at 0.5 eV at 100 K and 290 K, respectively, and (**e**,**g**) the difference in Δ*R*/*R* between double and single photoexcitation profiles. The second pump fluences are fixed at 9.0 mJ cm^−2^ at 100 K and at 4.8 mJ cm^−2^ at 290 K. (**h**) Calculated time profiles of low-energy spectral weight *D*(*t*) and (**i**) difference between double and single photoexcitation profiles in the insulating condition. The amplitude of the first pump-pulse vector potential, (*e*/*c*)*A*_1st_, was varied while that of the second pulse, (*e*/*c*)*A*_2nd_, was fixed at 0.6.

**Figure 5:**
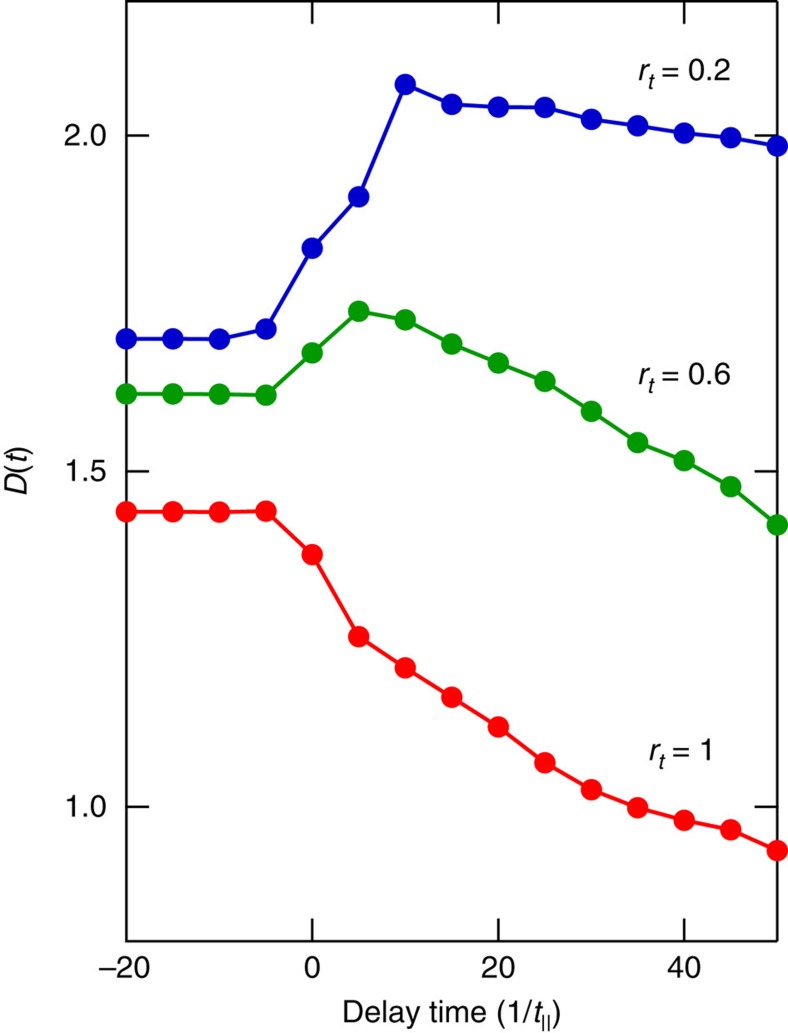
**Time profiles of low-energy spectral weights in metallic condition for several anisotropies of the hopping matrix elements** ***r***_***t***_**=*****t***_**⊥**_**/*****t***_‖_. The Drude weight *D*(*t*) is defined as an integrated spectral weight up to *ω*=2*t*_‖_. Red, green, and blue circles indicate the results at *r*_*t*_=1, 0.6 and 0.2, respectively.

**Figure 6:**
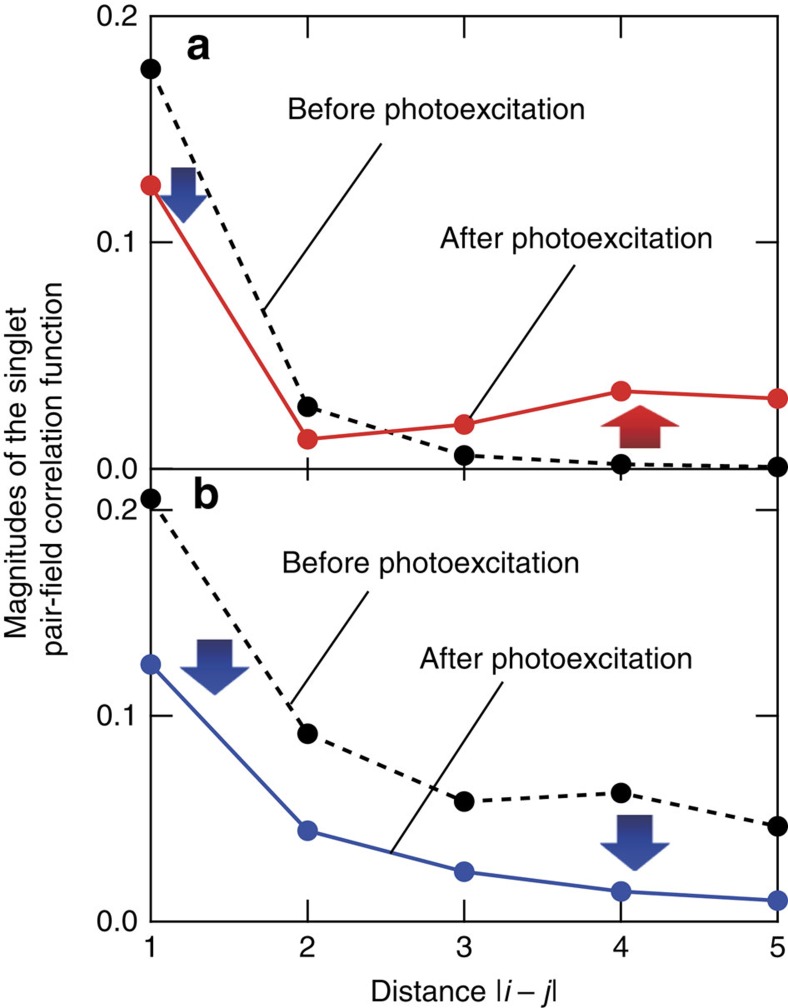
Transient singlet pair-field correlations in insulating and metallic states. (**a**,**b**) Calculated magnitudes of the singlet pair-field correlation functions before and after photoexcitation, which are respectively defined as |*P*(|*i*-*j*|, *t*<<0)| and as the time-averaged |*P*(|*i*-*j*|, *t*)| between *t*=20/*t*_‖_−50/*t*_‖_, for insulating (*n*=1) and metallic (*n*=0.833) conditions. A finite-sized cluster of 2 × 6 sites with open boundary conditions along the legs is used. The on-site Coulomb interaction is *U*/*t*_‖_=6. The electron hopping matrix elements *t*_‖_(legs) and *t*_⊥_(rungs) are an isotopic condition, *t*_⊥_/*t*_‖_. The photoexcitation simply decreases the magnitude of the correlation in the metallic condition at any distance (see the blue arrows in **b**). In contrast, magnitude of the correlation in the insulating condition increases at long distances (the red arrow in **a**).

Here we introduce the transient wave function Ψ(*t*), and the singlet pair-field Δ_*i=*_*c*_*i*2↓_*c*_*i*1↑_−*c*_*i*2↑_*c*_*i*1↓,_ where *c*_*iλσ*_ is the annihilation operator of a hole at rung *i* and the right (*λ*=1) or left (*λ*=2) leg with spin *σ*(=↑or↓). This function measures the correlation wherein a singlet hole pair is added to rung *i* and is removed at rung *j*. We observe the magnitudes of the pair-field correlations before and after photoexcitation. Figure 6a shows the magnitudes of the pair-field correlation as functions of the distance |*i*-*j*| for the insulating ladder. The photoexcitation enhances the magnitudes of the correlations at large distances, which is similar to the correlation in the metallic case before the photoexcitation (see the dashed line in Fig. 6b). In contrast, as shown by the blue line in the figure, the magnitude of the correlation in the metallic case is reduced for all distances and the pair correlation changes to short range, which is similar to the correlation in the insulating case before the photoexcitation (see the dashed line in Fig. 6a). The opposite changes in the pair-field correlations between the insulating and metallic cases correspond to the contrast photoinduced changes in the Drude weights shown in Supplementary Fig. 1b and Fig. 3c,d. We propose from these calculations a possible scenario that the photo-injected holes reduce the carrier-pair coherence in the metallic state, as is schematically shown in Fig. 1e, and suppress the metallicity of the system.

This study demonstrated for the first time that ultrafast I–M conversion at room temperature can be achieved by photo-tuning of the carrier-pair coherence in a Cu-ladder system that is known to be an unconventional superconductor. Supplementary Movie 1 is a detailed animation of this process, indicating that the metallicity in the system is suppressed in spite of the hole doping by the photoexcitation. The carrier conductive feature of photoinduced insulating state of SCCO is distinctly different from that of the chemically and thermodynamically accessible insulating state, and it can be ascribed to a hidden state^6^. The sequential photoexcitation results strongly suggest that the hole pairs exist even in the photoinduced metallic state under sufficiently high carrier density conditions and that the additional photoexcitation induces the collapse of hole-pair coherence. The theoretical model provided a clue to understand the unusual optical response in the metallic ladder cuprate, although full treatment of the relaxation process is a challenging issue for the next stage in near future research. These represent an important new degree of freedom for determining the nature of the carrier conductivity and it can be used to realize not only I to M but also M to I conversion by virtue of a photoinduced hidden insulating state over a wide temperature range including room temperature. Moreover, carrier-coherence control provides new insights into I–M conversion and the superconducting mechanism in correlated electron systems. It has opened a new window on non-equilibrium materials science and is promising for ultrafast room-temperature-operated phase switching.

## Methods

### Sample preparation

Single crystals of Sr_14_Cu_24_O_41_ and Sr_4_Ca_10_Cu_24_O_41_ were grown using the travelling-solvent-floating-zone method. Reflectivity spectra for each compound were obtained by using a Fourier transform interferometer (0.1–0.8 eV) and grating-type monochromator (0.7–5 eV).

### Femtosecond pump–probe reflection spectroscopy

The transient reflectivity change (Δ*R*/*R*) was measured using a pump–probe technique. A Ti:sapphire regenerative amplifier system (photon energy: 1.58 eV; pulse width: 120 fs; repetition rate: 1 kHz) was used as the light source. The optical pulse output from the amplifier system was divided into pump and probe pulses. The pump pulse was a fundamental of the output light, and the repetition rate was tuned to the half of the fundamental frequency (500 Hz) with an optical chopper. The probe pulse was converted into the 0.12–4.0 eV energy range using a frequency mixing process. Here optical parametric amplification was used in the range of 0.5–0.7 eV (idler light) and in the range of 0.8–1.0 eV (signal light), with differential frequency generation between the signal and idler light in the range of 0.12–0.4 eV, second harmonic generation of the idler light in the range of 1.1–1.5 eV and of the signal light in the range of 1.6–2.1 eV and fourth harmonic generation of the idler light in the range of 2.2–3.0 eV and of the signal light in the range of 3.1–4.0 eV. The typical probe pulse width was 150 fs. Δ*R*/*R* was measured using HgCdTe (0.12–0.2 eV), PbSe (0.3–0.5 eV), PbS (0.6–1.0 eV), Si (1.1–2.4 eV) and GaP (2.5–4.0 eV) detectors in the photon energy ranges of the probe light, and the signals were collected with a gated boxcar integrator. The samples were mounted in an optical helium cryostat, with both pump and probe beams entering the sample at near normal incidence and the positions of both beams at the sample were fixed in order to maintain similar experimental conditions at the various probe photon energies. The diameter of the pump light at the sample position was ca. 500 μm, which was about triple the diameter of the probe light. The polarizations of the pump and probe light were parallel to the *c* axis of the sample. In the sequential photoexcitation measurements, the pump pulse was divided with a first beam splitter, as shown in Supplementary Fig. 4. The interval and fluence between the two separate pulses were controlled by using a delay stage and a variable neutral density filter, respectively, and these pulses were recombined coaxially with the second beam splitter.

### Kramers–Kronig analysis on transient reflectivity spectrum

The Kramers–Kronig (K–K) analysis on the transient reflectivity spectrum takes into account of the spatially inhomogeneous distribution on the photoinduced part after the pumping^19^,^20^. After the photoexcitation, the intensity of the incident pulse exponentially decreases with the distance (*z*) from the surface, so that the photoexcited region also exponentially decays along *z*. Under these circumstance, the total dielectric function (*ɛ*^total^) in the photoexcited state is described by a linear combination of the dielectric constant in the photoexcited state (*ɛ*^PI^, unknown) and that in the original state (*ɛ*^O^, already known) as in the following;


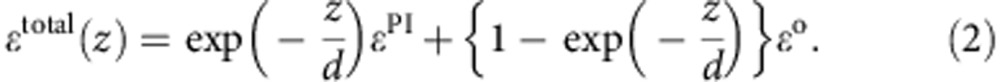


Here *d* is penetration depth of the pump light (∼80 nm). With this relation, the reflectivity (*R*) as well as the phase change (*θ*) at *z*=0 can be expressed as a function of ɛ^total^(*z*), or the real and imaginary part of *ɛ*^PI^.

We can calculate the actual value of *R* from Δ*R/R* (*R*_exp_) and *θ* in terms of K–K analysis (*θ*_exp_) after the photoexcitation. Thus we can construct the following simultaneous equations,


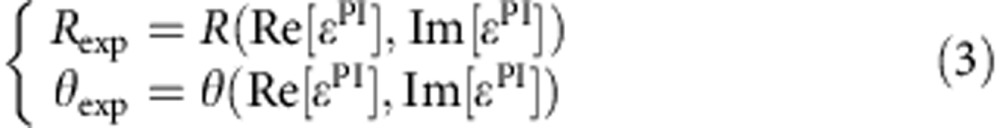


If we solve these nonlinear equations, we can obtain the real and imaginary part of the *ɛ*^PI^ and optical conductivity defined as *ω*Ιm[*ɛ*^PI^]/4*π* (*ω*: frequency). In the K–K analysis, we assumed constant reflectivity below 0.1 eV and connected to the obtained transient reflectivity with the reflectivity before the photoexcitation above 4.0 eV on the ground that the photoinduced reflectance change is quite small above 4.0 eV.

### Theoretical calculations

Real-time evolutions of photoexcited electronic states were calculated by applying the Lanczos algorithm-based exact-diagonalization method to finite-sized clusters. Insulating SCO and hole-doped metallic SCCO were modelled with a half-filled Hubbard ladder having an electron density *n=*1 and a metallic ladder (*n*=0.833), respectively. All times and energies were measured in terms of 1/*t*_‖_ and *t*_‖_, respectively, where 1/*t*_‖_ is equivalent to about 1 fs when we chose *t*_‖_=0.3–0.5 eV for ladder cuprates^25^. In most of the numerical calculations, we adopt *U*=6*t*_‖_ and *t*_‖_=*t*_⊥._ The obtained charge excitation gap in the insulating system is about 3.5*t*_‖_ (Supplementary Fig. 1b) that is a reasonable value for the CT excitation in the insulating SCO. The pump pulse was incorporated in the electron-transfer term in the Hamiltonian as the Peierls phase. The vector potential of the pump pulse was assumed to be of the form, *A*(*t*)=*A* cos(*ωt*) exp(−*t*/(2*γ*^2^)), with amplitude *A*, frequency *ω*, and damping factor *γ*. The center of the pump pulse was defined to be at time *t*=0. The polarization of the pump pulse was parallel to the leg direction. The numerical value of *γ* was chosen to be 1–5/*t*_‖_, and those of *ω* are indicated by bold inverted triangles in Fig. 3c,d. The transient electronic structures were monitored by simulating the pump–probe optical absorption spectra with a dynamical current-current correlation function for the time-dependent wave function |Ψ(*t*)>. Several other transient physical quantities, for example, kinetic energy, Coulomb interaction energy, singlet pair-correlation functions, electronic density of states and one-particle excitation spectra were also calculated.

## Additional information

**How to cite this article:** Fukaya, R. *et al*. Ultrafast electronic state conversion at room temperature utilizing hidden state in cuprate ladder system. *Nat. Commun.* 6:8519 doi: 10.1038/ncomms9519 (2015).

## Supplementary Material

Supplementary Information Supplementary Figures 1-4, Supplementary Table 1, Supplementary Notes 1-2 and Supplementary References

Supplementary Movie 1 "Animation of ultrafast metallicity suppression dynamics in SCCO" - Schematic animation based on the experimental and theoretical results of the hole-pair coherence control leading to the ultrafast suppression of metallicity in SCCO.
