# Supplementary material for: Ultrafast electronic state conversion at room temperature utilizing hidden state in cuprate ladder system
Source: Nat Commun. 2015 Oct 20;6:8519. doi: 10.1038/ncomms9519 (PMC4634131; doi:10.1038/ncomms9519)
Supplement: Supplementary Information — Supplementary Figures 1-4, Supplementary Table 1, Supplementary Notes 1-2 and Supplementary References [file ncomms9519-s1.pdf]

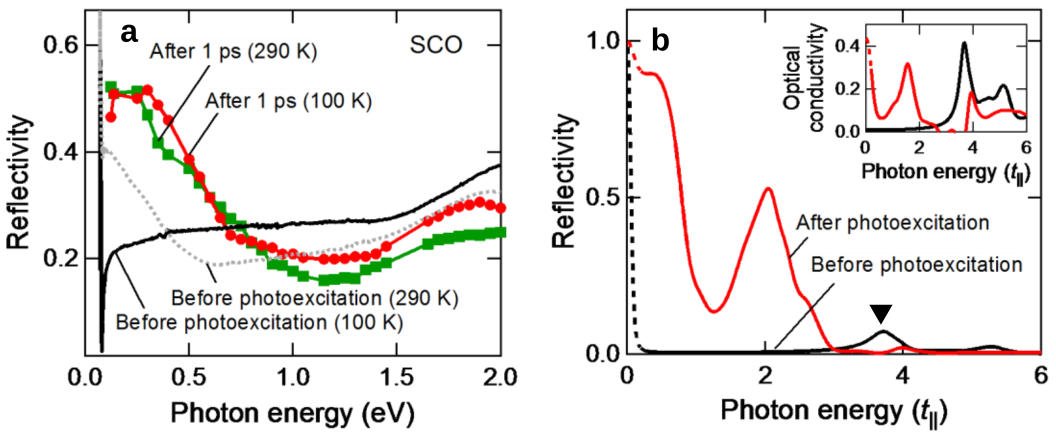

Supplementary Figure 1 | Reflectivity spectra before and after photoexcitation in SCO. (**a**) Reflectivity spectra with polarization of $E \| c$ before and after photoexcitation on SCO at 100 K (solid line and circles) and 290 K (dashed line and square)[1]. The excitation fluence is about 13 mJcm^{-2} at both temperatures. (**b**) Calculated reflectivity with polarization along the leg before ($t \ll 0$) and after ($t=20/t_{\parallel}$) photoexcitation under insulating condition (n=1). A finite-sized cluster of 2×6 sites with open boundary condition along the legs is used. The on-site Coulomb interaction is $U/t_{\parallel}=6$. Bold inverted triangle denotes the pump-photon energy. The inset of **b** indicates the calculated optical conductivity before (black) and after (red) the photoexcitation. Negative value in the spectrum after the photoexcitation implies optical emission. The collapse of the optical gap around 3.7 $t_{\parallel}$ and the appearance of a large low-energy component, corresponding to the Drude response in the finite-sized cluster calculations[2], imply a photoinduced I-to-M conversion. A peak structure around 2 $t_{\parallel}$ appearing after photoexcitation is attributed to the incoherent absorption component originating from coupling between charge carriers and spin fluctuations. The dashed lines below 0.2 $t_{\parallel}$ corresponds to reference data due to the calculation size effect.

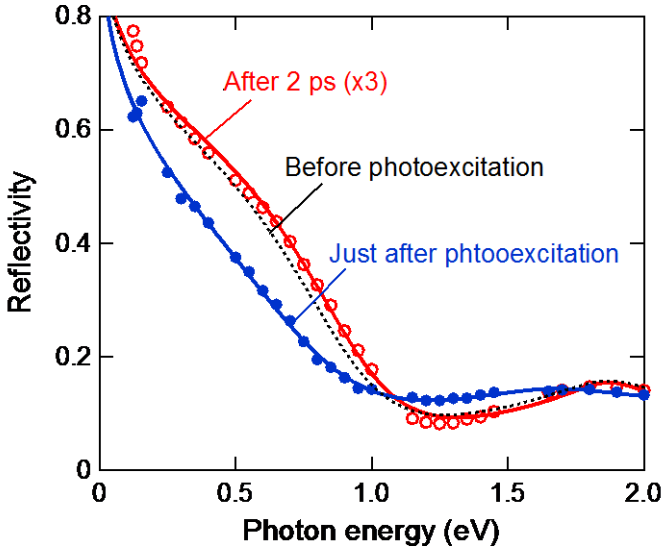

Supplementary Figure 2 | Transient reflectivity spectra at characteristic delay time SCCO at room temperature. The data just after the photoexcitation, plotted by filled blue circles, is the same as that shown in Fig. 3a in the main text. The open red circles denote the spectra after 2 ps. The black dotted line and blue and red solid lines indicate the curve fittings using the Drude-Lorentz model described in Supplementary Note 1 before the photoexcitation, just after the photoexcitation, and after 2 ps, respectively. The fitting parameters are summarized in Supplementary Table 1. The low-energy reflectivity reduces just after the photoexcitation, which is attributed to the suppression of the Drude weight discussed in the main text. It should be noted that the reflectivity at 2 ps is larger below 1.1 eV and smaller above 1.1 eV than that before the photoexcitation, strongly indicates that the Drude weight at 2 ps is qualitatively larger than that before the photoexcitation. This clearly indicates that the first reduction response is driven not by a simple heating effect but by a photonic change of the electronic state, because the second enhanced response cannot be viewed as a thermal relaxation process and is consistent with the enhancement of metallicity that in SCO.

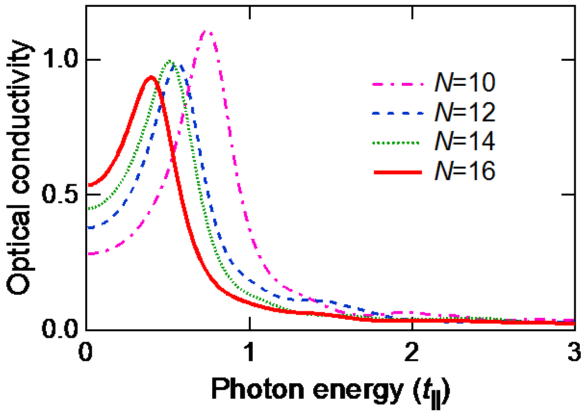

Supplementary Figure 3 | Size dependence of the calculated optical conductivity spectra. The optical conductivity spectra in the ground state for hole-doped cases are calculated in finite-sized clusters with open boundary conditions along the leg direction. N is the number of Cu sites in the cluster. The numerical values of U and $t_{\perp}$ were chosen to be 6 and 1, respectively, and the number of holes was fixed at 2.

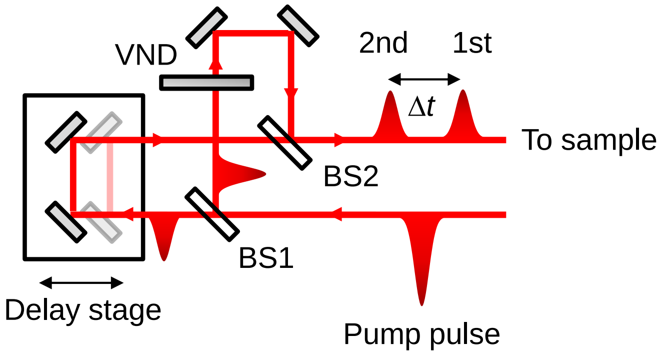

Supplementary Figure 4 | Schematic diagram of the optics to create sequential pulses. The pump pulses were divided with a first beam splitter (BS1). The interval (Δt) and fluence between two separate pump pulses were controlled by using a delay stage and a variable neutral density filter (VND), respectively. These pulses were reproduced coaxially with the second beam splitter (BS2).

Supplementary Table 1 | Parameters of fitting analysis of transient reflectivity spectra in SCCO. Fitting parameters of Drude weight (*D*) and scattering rate (Γ) before the photoexcitation, just after the photoexcitation (0 ps) and after 0.2 and 2 ps, obtained using the Drude-Lorentz model (Supplementary Equation 1).

Delay time	Before	0 ps	0.2 ps	2 ps
D	0.147	0.132	0.153	0.150
Γ (eV^{-1})	0.634	0.868	0.637	0.610

Supplementary Note 1 | Analysis of transient reflectivity spectra in SCCO

To quantitatively evaluate the optical parameters in the photoexcited state of SCCO, we directly analyzed the transient reflectivity in terms of the Drude-Lorentz model:

$$\varepsilon(\omega) = \varepsilon_\infty - \frac{\omega_p^2}{\omega(\omega - i\Gamma)} + \sum_j^2 \frac{S_j \omega_j^2}{\omega(\omega_j - \omega + i\Gamma_j)}, \tag{1}$$

where ε_∞ is the dielectric constant at the high-frequency limit, ω_p is the plasma frequency, Γ is the damping frequency of the Drude component, and Γ_j, S_j, and ω_j are the damping frequency, the oscillator strength, and the peak frequency of each CT components, respectively. In the ladder system, two CT components exhibit at 1.8 and 2.7 eV, which are assigned to the CT transition from the O 2*p* band to the Cu 3*d* upper Hubbard band in the ladder and chain sublattices, respectively[3,4]. As shown by the fitting curves in Supplementary Fig. 2, the reflectivity spectra was well reproduced by Supplementary Equation (1). The value of ω_p^2 is proportional to the Drude weight (D=[$(m_0\varepsilon_0)/\hbar^2N$]$\omega_p^2$; m_0: free electrom mass; ε_0: dielectric constant in a vacuum; N: number of Cu atoms in the unit cell volume), which is proportional to n/m^* (n: number of holes per Cu site; m^*; effective mass divided by the bare mass of an electron), and we can numerically estimate the time development of D from ω_p^2 obtained by the fitting analysis. The values of D and Γ at the characteristic delay times are summarized in Supplementary Table 1. The value of D before the photoexciation was greatly reduced and that of Γ increased immediately afterwards. It should be noted that the reduced D was recovered and increased by about 4 % while Γ recovers at 0.2 ps. With further time elapsed, Γ is reduced below that in the original state at 2 ps. These analyses clearly indicate that the first reduction response is driven not by a simple heating effect but by a photonic change of the electronic state, because the second enhanced response cannot be viewed as a thermal relaxation process and is consistent with the enhancement of metallicity that in SCO.

Supplementary Note 2 | Relationship of carrier density between photo and chemical doping in SCO

In the previous work[1], we quantitatively revealed the relationship between photo and chemical doping by analyzing the fluence dependence of the Drude weight. As reported in the optical study by Osafune et al.[3], the carrier density estimated from the low-energy spectral weight increases linearly with increasing Ca composition, i.e. chemical doping. In this framework, the low-energy spectral weight can be regarded as the increased value of the Drude weight from the initial state. According this estimation, the pumping at about 8 mJ/cm^2 , as shown in the vertical dashed line in Figs. 4b, c, creates the metallic state where the carrier density is comparable to the $Sr_5Ca_9Cu_{24}O_{41}$ compound on the phase boundary of the M-I transition[5,6].

Supplementary References

1. Fukaya, R. *et al*. Unconventional photonic change of charge-density-wave phase in two-leg ladder cuprate $Sr_{14}Cu_{24}O_{41}$. *J. Phys. Soc. Jpn*. **82**, 083707 (2013).
2. Wagner, J., Hanke, W. & Scalapino, D. J. Optical, magnetic, and single-particle excitations in the multiband Hubbard model for cuprate superconductors. *Phys. Rev. B* **43**, 10517-10529 (1991).
3. Osafune, T. *et al*. Optical study of the $Sr_{14-x}Ca_xCu_{24}O_{41}$ sytem: evidence for hole-doped Cu_2O_3 ladders. *Phys. Rev. Lett*. **78**, 1980-1983 (1997).
4. Popović, Z. V. *et al*. Optical properties of the spin-ladder compound $Sr_{14}Cu_{24}O_{41}$. *Phys. Rev. B* **62**, 4963-4972 (2000).
5. Kojima, K.M., Motoyama, N., Eisaki, H. & Uchida, S., The electronic properties of cuprate ladder materials. *J. Electron Spectrosc. Relat. Phenom.* **117**, 237 (2001).
6. Vuletić, T. *et al*., The spin-ladder and spin-chain system $(La,Y,Sr,Ca)_{14}Cu_{24}O_{41}$: Electronic phases, charge and spin dynamics, *Phys. Rep.* **428**, 169 (2006).
